# Supplementary material for: A whole-animal phenotypic drug screen identifies suppressors of atherogenic lipoproteins
Source: eLife. 2026 Aug 27;14:RP105314. doi: 10.7554/eLife.105314 (PMC13521566; doi:10.7554/eLife.105314)
Supplement: Supplementary file 2 [file elife-105314-code1.zip › JHDL_primary_result/RESERPINE/RESERPINE_JHDL_7_018_008_2019_06_07_12_15.pdf]

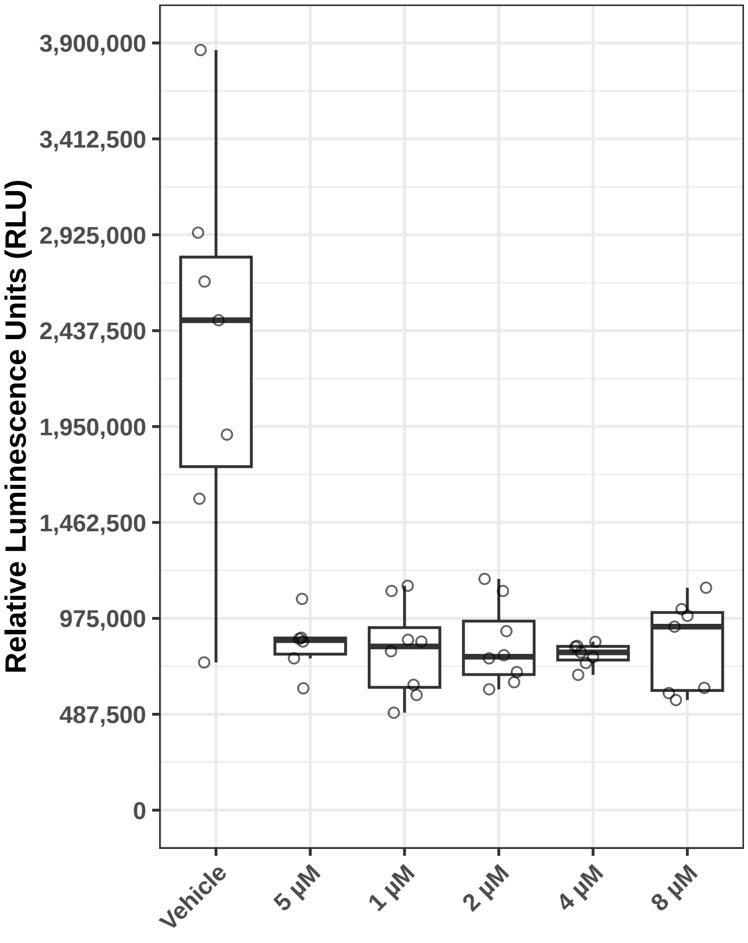
Relative Luminescence Units (RLU)
3,900,000
3,412,500
2,925,000
2,437,500
1,950,000
1,462,500
975,000
487,500
0
Vehicle
5 μM
1 μM
2 μM
4 μM
8 μM
